# Metabolic factors and the risk of Dupuytren’s disease: data from 30,000 individuals followed for over 20 years

**DOI:** 10.1038/s41598-021-94025-7

**Published:** 2021-07-19

**Authors:** Mattias Rydberg, Malin Zimmerman, Jin Persson Löfgren, Anders Gottsäter, Peter M. Nilsson, Olle Melander, Lars B. Dahlin

**Affiliations:** 1 Department of Hand Surgery, Lund University, Skåne University Hospital, Jan Waldenströms gata 5, 205 02 Malmö, Sweden; 2 Department of Translational Medicine-Hand Surgery, Lund University, Lund, Sweden; 3 Department of Vascular Diseases, Lund University, Skåne University Hospital, Malmö, Sweden; 4 Department of Emergency and Internal Medicine, Skåne University Hospital, Malmö, Sweden; 5 Department of Clinical Sciences, Lund University, Malmö, Sweden

**Keywords:** Epidemiology, Diabetes complications, Connective tissue diseases, Musculoskeletal abnormalities

## Abstract

Dupuytren’s disease (DD) is a fibroproliferative disorder affecting the palmar fascia of the hand. Risk factors include diabetes mellitus (DM), whereas a high body mass index (BMI) is associated with a lower prevalence of DD. The aim of this study was to further elucidate risk and protective factors for the development of DD using longitudinal population-based data from the Malmö Diet and Cancer Study (MDCS). During 1991–1996, the inhabitants aged 46–73 years in the city of Malmö, Sweden were invited to participate in the population-based MDCS (41% participation rate). Data on incident DD were retrieved from Swedish national registers. Associations between DM, alcohol consumption, BMI, and serum apolipoprotein A1 (ApoA1) and apolipoprotein B (ApoB) at baseline were analysed in multivariable Cox regression models adjusted for known confounders. Among 30,446 recruited participants, 347 men and 194 women were diagnosed with DD during a median follow-up time of 23 years. DM (men HR 2.23; 95% CI 1.50–3.30, women HR 2.69; 95% CI 1.48–4.90) and alcohol consumption (men HR 2.46; 95% CI 1.85–3.27, women HR 3.56; 95% CI 1.95–6.50) were independently associated with incident DD in the Cox regression models. Furthermore, inverse associations with incident DD were found for obesity among men, and ApoB/ApoA1 ratio among both sexes. DM and excess alcohol consumption constituted major risk factors for the development of DD. Furthermore, an inverse association between obesity among men and DD, and also between ApoB/ApoA1 ratio and DD was found in both sexes.

## Introduction

Dupuytren’s disease (DD) is a fibroproliferative disorder affecting the palmar fascia of the hand. It often starts with nodules in the hand that over time thickens and form cords, contracting the finger and thus causing Dupuytren’s Contracture. Although any finger can be affected, DD is most often diagnosed in the ring and middle fingers^1^. The prevalence of DD varies significantly in the literature, depending on the region and population studied^2,3^. A recent Swedish large-scale population-study found a 1.35% prevalence of DD among men and 0.5% among women^4^. Treatment options include pharmacological injection of collagenase, needle fasciotomy, and open fasciectomy^1^.

The exact aetiology and pathogenesis of DD is still debated although several risk factors have been proposed, including smoking^5^, alcohol consumption^6^, hypertension^7^, manual work^8^, genetic susceptibility^9^, and diabetes mellitus (DM)^10^. Moreover, different body anthropometrics, including body mass index (BMI), have been investigated in relation with DD with relatively consistent results, indicating lower risk for DD with higher BMI^11,12^. Interestingly, recent genetic studies have proposed a negative correlation between gene variants for increased BMI and DD, supporting an inverse association between BMI and DD^13^. Finally, previous studies have associated DD with increased serum cholesterol and triglyceride levels^14^ and also with alterations in lipid deposition in the palmar fat tissue^15^. However, the number of longitudinal cohort studies in the DD field in general is low^16^, and studies aiming to control for potential confounding factors are lacking. Therefore, the *aim* of this observational study was to use longitudinal data from a population-based cohort study, the Malmö Diet and Cancer study (MDCS)^17^, in order to explore associations between DD and baseline DM, BMI, alcohol consumption, and apolipoprotein levels during long-term follow-up.

## Results

### Baseline characteristics

**Figure 1:**
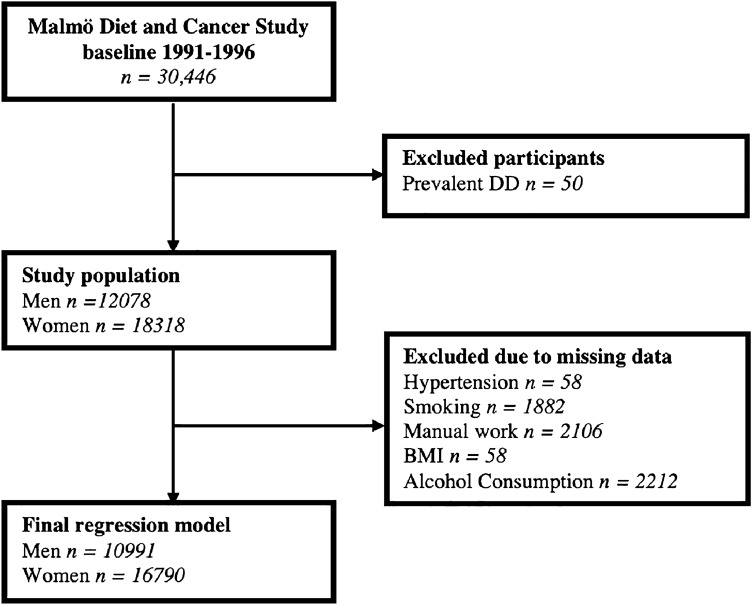
Derivation of the study cohort from the Malmö Diet and Cancer Study (MDCS). *BMI* body mass index, *DD* Dupuytren’s disease.

Baseline characteristics, stratified by sex, are presented in Table 1. Baseline characteristics stratified by incident DD and sex are presented in Table 2a,b. Fifty participants were excluded from further analysis due to a prevalent diagnosis of DD at baseline (Fig. 1). In total, there were 541 participants diagnosed with DD during a median follow-up of 23 [IQR 15.8–30.2] years; 347 men and 194 women. This corresponds to an incidence of 1.47 cases per 1000-person years for men, and 0.49 cases per 1000 person-years for women. Mean age at diagnosis was 71 years for both men (SD 7.0) and women (SD 7.5).

**Table 1:** Baseline characteristics stratified for sex. Normal weight; BMI < 25, overweight; BMI ≥ 25 to < 30, obese BMI ≥ 30 kg/m^2^. Low alcohol consumption < 15 g /day, Moderate alcohol consumption 15–30 g/day, Heavy alcohol consumption > 30 g/day. Participants with prevalent DD at baseline (n = 50) were excluded. *DD* Dupuytren’s disease, *g* grams, *SD* standard deviation.

Characteristics	Women (n = 18,318)	Men (n = 12,078)
Age, years (mean ± SD)	56.8 ± 7.9	58.7 ± 7.0
**BMI (kg/m**^**2**^**) (mean ± SD)**	25.5 ± 4.3	26.3 ± 3.5
Normal weight (n [%])	9591 (52.5)	4488 (37.2)
Overweight (n [%])	6088 (33.3)	5932 (49.2)
Obesity (n [%])	2606 (14.3)	1633 (13.5)
Current smoking (n [%])	4858 (28.1)	3218 (28.7)
Hypertension (n [%])	7712 (42.2)	6437 (53.4)
Low alcohol consumption (n [%])	14,303 (83.7)	6661 (60)
Moderate alcohol consumption (n [%])	2388 (14.0)	2850 (25.7)
Heavy alcohol consumption (n [%])	402 (2.4)	1585 (14.3)
Diabetes mellitus (n [%])	656 (3.6)	742 (6.1)
Manual work (n [%])	6594 (38.5)	4054 (36.3)
Apolipoprotein A1, g/L (mean ± SD)	1.64 ± 0.3	1.45 ± 0.2
Apolipoprotein B, g/L (mean ± SD)	1.05 ± 0.3	1.11 ± 0.3
Incident DD (n [%])	194 (1.1)	347 (2.9)
Incidence rate DD (cases/1000 person-years)	0.49	1.47

**Table 2:** a, b Baseline characteristics with all subjects, stratified with regard to incident **DD**.

(a) Male participants	No DD n = 11,731	Incident DD n = 347	p value*
Age, years (mean ± SD)	58.6 ± 7.0	56.8 ± 6.4	**< 0.001**
BMI (kg/m^2^) (mean ± SD)	26.3 ± 3.6	25.9 ± 3.3	**< 0.028**
Current smoking (n [%])	3118 (28.7)	100 (30.5)	= 0.47
Hypertension (n [%])	6275 (53.6)	162 (46.7)	**= 0.01**
Diabetes mellitus (n [%])	713 (6.1)	29 (8.4)	= 0.08
Alcohol consumption g/day [IQR]	11.2 [2.0–20.3]	17.2 [6.9–27.5]	**< 0.001**
Manual work (n [%])	3942 (36.4)	112 (34.3)	= 0.43
ApoA1, g/L (mean ± SD)	1.45 ± 0.2	1.52 ± 0.3	**< 0.001**
ApoB g/L (mean ± SD)	1.11 ± 0.3	1.10 ± 0.3	= 0.5
ApoB/ApoA1 ratio (mean ± SD)	0.78 ± 0.2	0.74 ± 0.2	**< 0.001**

(b) Female participants	No DD n = 18,124	Incident DD n = 194	p value*
Age, years (mean ± SD)	56.8 ± 7.9	56.1 ± 7.0	= 0.18
BMI (kg/m^2^) (mean ± SD)	25.5 ± 4.3	25.2 ± 3.9	= 0.26
Current smoking (n [%])	4811 (28.1)	47 (25.3)	= 0.4
Hypertension (n [%])	7644 (42.3)	68 (35.2)	**= 0.049**
Diabetes mellitus (n [%])	643 (3.5)	13 (6.7)	**= 0.019**
Alcohol consumption g/day [IQR]	5.4 [0.1–10.7]	6.9 [0.2–13.6]	**= 0.01**
Manual work (n [%])	6523 (38.5)	71 (38.6)	= 0.97
ApoA1, g/L (mean ± SD)	1.64 ± 0.3	1.71 ± 0.3	**= 0.01**
ApoB g/L (mean ± SD)	1.05 ± 0.3	1.00 ± 0.2	**= 0.01**
ApoB/ApoA1 ratio (mean ± SD)	0.66 ± 0.2	0.61 ± 0.2	**= 0.003**

Participants with prevalent DD at baseline (n = 50) were excluded.

*P-value for group comparison between subjects without DD and with incident DD; independent sample t-test used for age, BMI and lipid levels, Chi-Square test for dichotomous variables and Mann–Whitney U test was used for alcohol consumption. Participants with prevalent DD are excluded. Bold values indicating *p* < 0.05.

*APO* Apolipoprotein, *BMI* body mass index, *DD* Dupuytren’s disease, *IQR* interquartile range, *SD* standard deviation.

### Incident DD by diabetes status, BMI and alcohol consumption

In total, there were 656 female and 742 male participants with prevalent DM at baseline (Table 1).

In the first sex-stratified Cox regression model, including only age and respective variable, prevalent DM at baseline was independently associated with DD among both men (HR 2.03; 95% CI 1.38–3.00; p < 0.001) and women (HR 2.38; 95% CI 1.32–4.29; p = 0.004). When including all variables, i.e. age, BMI-group, alcohol consumption, smoking status, manual work and hypertension, in the multivariable Cox regression model, prevalent DM was still independently associated with incident DD among both men (HR 2.23; 95% CI 1.50–3.30; p < 0.001) and women (HR 2.69; 95% CI 1.48–4.90). Furthermore, among both men and women, there was a significant association between both moderate and heavy alcohol consumption and incident DD in both Cox regression models. Finally, in the multivariable model, there was an inverse association between obesity and incident DD among men (HR 0.66; 95% CI 0.44–0.98 p = 0.04). Among women, there was a borderline significant inverse association between obesity and DD (HR 0.55; 95% CI 0.33–1.00 p = 0.05) (Table 3). No significant associations were found between smoking, hypertension, overweight, and incident DD, neither among men nor women.

**Table 3:** Sex-stratified Cox regression models with HR for incident DD in relation covariates.

	Model I*		Model II**	
	HR (95% CI)	p-value	HR (95% CI)	p-value
**Men**				
Diabetes mellitus	2.03 (1.38–3.00)	**< 0.001**	2.23 (1.50–3.30)	**< 0.001**
Current smoking	1.19 (0.94–1.52)	= 0.15	1.10 (0.86–1.40)	= 0.43
Hypertension	0.93 (0.74–1.17)	0.54	0.90 (0.72–1.14)	= 0.38
Low alcohol consumption	*Reference*	–	*Reference*	–
Moderate alcohol consumption	1.62 (1.26–2.10)	**< 0.001**	1.67 (1.29–2.16)	**< 0.001**
Heavy alcohol consumption	2.37 (1.79–3.14)	**< 0.001**	2.46 (1.85–3.27)	**< 0.001**
Normal weight	*Reference*	–	*Reference*	–
Overweight	0.88 (0.70–1.11)	= 0.27	0.85 (0.67–1.07)	= 0.17
Obesity	0.70 (0.47–1.05)	= 0.08	0.66 (0.44–0.98)	**= 0.04**
Manual work	1.00 (0.80–1.26)	= 0.98	1.11 (0.98–1.02)	= 0.38
**Women**				
Diabetes mellitus	2.38 (1.32–4.29)	**= 0.004**	2.69 (1.48–4.90)	**= 0.001**
Current smoking	0.94 (0.67–1.32)	= 0.73	0.86 (0.61–1.22)	= 0.40
Hypertension	0.88 (0.64–1.21)	= 0.43	0.88 (0.64–1.12)	= 0.43
Low alcohol consumption	*Reference*	–	*Reference*	–
Moderate alcohol consumption	1.68 (1.17–2.42)	**= 0.005**	1.75 (1.20–2.53)	**= 0.003**
Heavy alcohol consumption	3.35 (1.85–6.07)	**< 0.001**	3.56 (1.95–6.50)	**< 0.001**
Normal weight	*Reference*	–	*Reference*	–
Overweight	1.07 (0.78–1.47)	= 0.66	1.04 (0.76–1.43)	= 0.80
Obesity	0.61 (0.36–1.06)	= 0.08	0.57 (0.33–1.00)	= 0.05
Manual work	1.06 (0.78–1.43)	= 0.71	1.21 (0.89–1.66)	= 0.23

*CI* confidence interval, *DD* Dupuytren’s disease, *DM* Diabetes Mellitus, *HR* Hazard Ratio. Bold values indicating *p* < 0.05.

*Cox regression model including only age at study entry and respective variable.

**Including age, DM, hypertension, smoking, alcohol consumption group, weight group, and manual work.

### Sensitivity analysis and interaction analysis

In the sensitivity analyses, the first excluding 1303 participants with a BMI < 20.0 and the second excluding 7956 participants with a follow-up time > 25 years in the multivariable Cox regression model, the associations found between DM, alcohol consumption, obesity among men and DD remained unchanged (Supplementary Tables 1 and 2). No significant interactions were found between DM and BMI (p for interaction; men p = 0.42, women p = 0.82) or DM and alcohol consumption (p for interaction; men p = 0.59, women p = 0.73).

Finally, the associations between DM and DD remained unchanged when analyzing separate HRs for low and moderate to high alcohol consumers among both men and women (data not shown).

### Incident DD by apolipoprotein levels

Table 2a,b display sex-stratified distributions of ApoA1 and ApoB, respectively. ApoA1 was higher at baseline in participants with incident DD. ApoB among women and the ApoB/ApoA1 ratio among both men and women were lower in participants with incident DD.

In the fully adjusted Cox regression model, one SD increment of ApoA1 was associated with DD in both men (HR_per SD_ 1.22; 95% CI 1.09–1.38; p < 0.01) and women (HR_per SD_ 1.17; 95% CI 1.01–1.36; p = 0.03). ApoB was inversely associated with DD among women (HR_per SD_ 0.83; 95% CI 0.70–0.99; p = 0.03), however no significant association was found among men. Finally, the ApoB/ApoA1 ratio was inversely associated with incident DD in both men (HR_per SD_ 0.85; 95% CI 0.75–0.96; p = 0.01) and women (HR_per SD_ 0.80; 95% CI 0.66–0.97; p = 0.02) (Table 4).

**Table 4:** Sex-stratified, multivariable Cox regression analysis with HR for incident DD in relation to ApoA1, ApoB and the ApoB/ApoA1 ratio, adjusted for age at baseline, hypertension, DM, alcohol consumption, BMI, manual work, and smoking.

Variable	HR (95% CI)^a^	p-value
**Men**		
ApoA1	1.22 (1.09–1.38)	**< 0.01**
ApoB	0.97 (0.86–1.09)	= 0.64
ApoB/ApoA1	0.85 (0.75–0.96)	**= 0.01**
**Women**		
ApoA1	1.17 (1.01–1.36)	**= 0.03**
ApoB	0.83 (0.70–0.99)	**= 0.03**
ApoB/ApoA1	0.80 (0.66–0.97)	**= 0.02**

*BMI* body mass index, *CI* confidence interval, *DD* Dupuytren’s disease, *HR* Hazard Ratio. Bold values indicating *p* < 0.05.

^a^HR are expressed as per one SD increase of respective Z-score converted variable.

## Discussion

The findings from this longitudinal population-based study with a median follow up of 23 years corroborate that DM and excessive alcohol consumption constitute major risk factors for the development of DD. Furthermore, we found an inverse association between obesity among men and DD. We also present data proposing an inverse association between ApoB/ApoA1 ratio and DD, findings that has not been previously reported. In addition to information regarding risk factors for DD, the study sheds light on the complex associations between DD, DM, and high BMI.

### Diabetes Mellitus

DM has long been considered a risk factor for DD, although the exact mechanisms by which hyperglycaemia affects the palmar fascia are still not fully understood^1,10^. One of the proposed biochemical processes underlying diabetic complications is the formation of advanced glycated end products (AGEs)^18^. AGEs have been associated with other fibroproliferative disorders, such as diabetic cardiomyopathy^19^ and idiopathic pulmonary fibrosis^20^, but also to complications of diabetes in the hand, e.g. carpal tunnel syndrome^21^. Indeed, data from biopsies from the palmar fascia in patients with DD showed higher levels of AGEs compared to a control group^22^, possibly increasing levels of collagen deposition and increased collagen stiffness^10,22^. Furthermore, previous studies have shown that DM increases the formation of myofibroblasts^23^, one of the proposed main cell types responsible for contraction the finger in DD^24^. Thus, future studies should examine the palmar fascia in patients with DM to clarify the effects of hyperglycaemia and AGEs on the development of DD. In our study, participants with prevalent DM at baseline had marked increased risk for development of DD during follow-up; even when adjusting for other known risk factors, e.g. alcohol consumption and BMI. Our results are in line with a previous meta-analysis on the effect of DM on the development of DD^10^. Our study adds important information due to its longitudinal setting and long follow-up, why it contributes to the establishment of DM as a major risk factor for the development of DD. Finally, our findings support the theory of DD as being a part of the more complex syndrome—“the diabetic hand”, together with diagnoses, such as carpal tunnel syndrome, trigger finger, and ulnar neuropathy^25–27^.

### Alcohol consumption and manual work

In our study, excessive alcohol consumers of both sexes had a marked higher risk of developing DD during follow-up, and these results are in line with several previous studies linking alcohol overconsumption to the development of DD^5,6^. Alcohol guidelines and thresholds for risk consumption differ notably from country to country^28^, but our study shows that already a somewhat moderate alcohol consumption (1–2 standard drinks per day), well below the definition of low-risk consumption in some countries^29^, is associated with an increased risk of DD. By adding long term follow-up data, our study strengthens the previously reported associations, establishing even a relatively low alcohol overconsumption as a major risk factor for developing DD. Furthermore, several previous studies have proposed manual work as a risk factor for the development of DD^8^, possibly through repeated microtrauma to the hand^30^. We found, however, no such associations in our study, neither among men or women, possibly due to the low number of farmers in the study population.

### BMI and apolipoproteins

The epidemiological literature on circulating lipids, apolipoproteins and risk of DD is scarce, and the most cited papers date back to the 1980’s and 1990’s^14,15^. In recent years, however, an inverse association between a high BMI and the risk of DD have been reported in several studies^11,12,31^, indicating a possible protective factor linked to the fat tissue. The putative biological mechanisms behind this associations is not known but lower serum testosterone among obese individuals has been proposed as one hormonal factor of importance, possibly decreasing the risk for DD^31^. In our study, we found a similar pattern of associations; men with incident DD during follow-up had a lower mean BMI at baseline compared to men without DD. Additionally, obese men had lower risk of DD compared to men with normal weight in the adjusted regression model, although the same results could not be convincingly shown among women.

However, we did find a strong association among both men and women between apolipoprotein levels and incident DD. Participants with a high ApoB/ApoA1 ratio had a lower risk of DD even when adjusting for age, DM, alcohol consumption, and BMI. Traditionally, the ApoB/ApoA1 ratio has been used as a predictor of cardiovascular disease^32,33^ and analysis of ApoB is still recommended as a complement or alternative to LDL-C in risk assessment of cardiovascular disease^34^. Our study is to the best of our knowledge the first to analyze apolipoprotein levels in relation to DD, why further studies are warranted, firstly to confirm these results, and secondly to better understand the biochemical background to the reported associations. Interestingly, recent genetic studies have proposed a negative genetic correlation between high BMI and DD^13^, supporting the previous results associating lower BMI with DD^11,12^. Furthermore, in studies on over 30,000 Danish twin pairs, the hereditability of DD was approximately 80%, whereas environmental risk factors, e.g. smoking or alcohol consumption, only contributes to 20% of the increased risk^9^. A recent Swiss study found that family history of DD was a strong risk factor for incident DD, emphasising the importance of genetic susceptibility for the development of the disease^35^. This could indicate that the associations found between apolipoproteins, low BMI and DD are due to genetic correlation rather than fat tissue being protective against DD. Since no genetic information was available in this study, we cannot draw any conclusions regarding such associations from our results, but it is an obvious target for future studies. Another future research target would be to examine palmar fascia biopsies from overweight and obese patients with and without DD, aiming to better understand the interplay between BMI, fat tissue, and DD risk.

To summarize, there might exist different protective and predisposing factors for the development of DD. Genetic predisposition, hormonal factors related to sex or high BMI, alterations in the palmar fascia due to DM and hyperglycemia, and finally, environmental factors such as alcohol consumption all interact in the complex pathogenesis behind DD (Supplementary Fig. S1).

### Strengths and limitations

One of the limitations of this study is the lack of standardized diagnostic criteria for DD during follow-up. The diagnoses were retrieved using ICD-codes and thus, physicians might have applied different criteria for diagnosis. Since we only used diagnoses from a specialized care setting, however, most diagnoses were established by trained specialists in hand or orthopedic surgery, strengthening case validity. Some participants might have been diagnosed with DD in primary care without specialist referral, which might have lowered our incidence rates to some extent. Indeed, data on incidence rates in our study are slightly lower than in a previous study from southern Sweden including data from primary care^4^, indicating that not all patients in Sweden with DD are referred to specialized care. Furthermore, since DD is a benign disease, some participants with moderate symptoms might not have sought healthcare at all despite having the disease and were thus not included in our registers. Taken together, our study design allowed us to analyze a clinically relevant diagnosis of DD, established in the specialized care setting, why results should be interpreted with this in mind. Another strength of this longitudinal study design is the lack of recall bias among participants, something that may otherwise be problematic in case–control studies.

Furthermore, a major strength of this study is the large population size, over 30,000 middle-aged individuals followed over 20 years making this the largest cohort study to date analyzing risk factors for DD. Moreover, the collection of baseline data was of high quality, with each participant being examined individually by a trained nurse. The data regarding prevalent DM are also of high quality, using several local and national registers for diagnosis acquirement. Unfortunately, we were not able to reliably stratify for type 1 and type 2 DM, making separate analyses for different types of DM a target for future research. Levels of glycemic control (HbA_1c_) in relation to DD risk would also be an interesting topic for research. Finally, a participant’s baseline characteristics, such as BMI and alcohol consumption, were only measured once. Undoubtably, these variables often change over a person’s lifetime, and this is a limitation that should be kept in mind when interpreting our results.

Even so, despite the high quality of baseline data and our adjustments for known confounders in the regression models, there is always a possibility that additional confounders might have influenced our results. With all this in mind, our conclusions are in line with several previous studies indicating both DM and excessive alcohol consumption as risk factors for the development of DD, thus making it unlikely that residual confounding would be sufficient to alter our main results significantly, but caution should always be taken when interpreting data from observational studies.

## Conclusion

DM and excess alcohol consumption constituted major risk factors for the development of DD in middle-aged subjects irrespective of sex. This study adds information regarding the debated pathogenesis underlying DD, but additional studies are warranted to further elucidate potential genetic markers for associations between DD, BMI and apolipoproteins.

## Methods

### Study population

A cohort from the MDCS^17^, consisting of 30,446 individuals from southern Sweden was studied. The participants living in the city of Malmö, born between 1926 and 1945 were invited to participate in the study between 1991 and 1996. Subjects underwent clinical examination, laboratory assessment and filled in a questionnaire regarding cardiovascular risk factors. The recruitment process has been previously described in detail^17^, the attendance rate was approximately 41%, and 60% of the participants were women.

### Baseline data collection

At baseline, a trained nurse measured participant’s height and weight. BMI was calculated as a participant’s weight/length^2^ (kg/m^2^) and divided into three categories; < 25 (normal weight), ≥ 25 to < 30 (overweight), and ≥ 30 (obese). Blood pressure (BP) was measured in a supine position with a mercury column sphygmomanometer, and hypertension was defined as a systolic BP ≥ 140 mmHg or diastolic BP ≥ 90 mmHg. Smoking was self-reported at baseline and defined as current smoker or non-smoker. Alcohol consumption was also self-reported as consumption during the last week in grams per day and divided into low consumers (< 15 g/day), moderate consumers (15–30 g/day) and heavy consumers (> 30 g/day). DM at baseline was defined as a fasting whole blood glucose > 7.0 mmol/L, a self-reported physician’s diagnosis or the use of antidiabetic medicine. Data on prevalent DM was also obtained using six other national and local registers as previously described in detail^36^. Data on current or latest occupation was based on self-reported job titles and categorized according to Swedish national population census^37^. Participants were classified as manual workers, which also included farming, and non-manual workers as previously described^38^.

At baseline, blood samples were collected from the participants and stored at − 80 °C. Levels of ApolipoproteinA1 (ApoA1) and Apolipoprotein B (ApoB) in serum were analysed by Quest Diagnostics (San Juan Capistrano, CA, USA) using an immunonephelometric assay run on a Siemens BNII (Siemens, Newark, DE, USA) in a total of 27,753 participants.

### Endpoints

Using Swedish national registers (Cause of Death Register, the Inpatient and Outpatient Registers) administrated by the National Board of Health and Welfare (http://socialstyrelsen.se/english), primary endpoint diagnosis, i.e. DD, were obtained by linkage of registers using each participants unique 10-digit personal number^39^. Using the International Classification of Disease (ICD) Swedish version 8, 9 or 10, both prevalent and incident diagnoses of DD were retrieved with the following ICD codes: 733.90/728G/M720. Only clinical, hospital-based diagnoses were used in this study, operation codes were not included. As the registers did not include diagnoses from primary health care, the study only includes diagnoses established in the specialized care mainly by specialists in hand- or orthopaedic surgery. Participants were followed from baseline until a diagnosis of DD in either of the registers, emigration, death, or end of study 2018-12-31, thus creating a time-to-event variable unique for each participant. Participants with a diagnosis of DD prior to baseline examination were excluded from further study.

### Statistical analysis

Baseline quantitative data were presented as mean with standard deviation (SD) when normally distributed and as median with interquartile range [IQR] when data was skewed. Count and proportion were used for presentation of nominal data. For group comparisons, independent sample t-test was used for age, BMI, ApoA1 and ApoB levels, Chi-Square test for dichotomous variables and Mann–Whitney U test was used for alcohol consumption. Levels of ApoA1, ApoB and the ApoB/ApoA1 ratio were analysed after a z-score standardization ([Variable level − mean]/standard deviation) resulting in a z-score variable with a mean of 0 and a SD of 1.

Potential associations between DD and prevalent DM, apolipoprotein levels, BMI, manual work and alcohol consumption were analyzed in Cox proportional hazards models stratified by sex. Two models were used; the first model included only age and the respective variable. The second multivariable model included BMI group, smoking habits, alcohol consumption, prevalent diabetes at baseline, hypertension, and manual work; i.e. the studied variable and possible confounders^5,7,10,11^. Hazard ratios (HR) with 95% confidence intervals (CI) were expressed with participants without DM at baseline as a reference when analysing DM, BMI < 25 kg/m^2^ as reference when analysing BMI groups, and low alcohol consumers as reference when analysing alcohol consumption. For ApoA1, ApoB and ApoB/ApoA1 ratio, HR with 95% CI were expressed as per one SD increment of each z-score converted variable.

To test for potential interaction between DM, alcohol consumption, and BMI, interaction variables (DM × Alcohol) and (DM × BMI) were introduced on top of the sex stratified, age-adjusted Cox regression models and analyzed, using continuous variables for alcohol consumption and BMI.

Finally, two sensitivity analyses were performed. The first one excluded participants with BMI < 20 in the multivariable Cox regression model, and the second one excluded participants with a follow-up time ≥ 25 years.

The assumption of proportional hazard was assessed by visual assessment of Kaplan–Meier curves and log–log plots and no violation was found. The statistical analyses were conducted using SPSS for Mac version 25 (SPSS Inc., Chicago, IL, USA). A two-tailed *p* value < 0.05 was considered significant.

### Ethics approval

The participants provided informed consent and both the MDCS and the present study were approved by the ethical committee at Lund University (DNR: LU51-90; 2009-633; 2019-01439). The study was conducted in accordance with the Helsinki Declaration.

## Supplementary Information

Supplementary Information.

## Data Availability

The data used for this study can be applied for by contacting the Steering Committee of the Malmö Diet and Cancer Study; (data manager Anders Dahlin, email: anders.dahlin@med.lu.se). For details of MDCS and how to apply for data, see web site: www.malmo-kohorter.lu.se/english.
