# Supplementary material for: Metabolic factors and the risk of Dupuytren’s disease: data from 30,000 individuals followed for over 20 years
Source: Sci Rep. 2021 Jul 19;11:14669. doi: 10.1038/s41598-021-94025-7 (PMC8289914; doi:10.1038/s41598-021-94025-7)
Supplement: Supplementary file 1 — Supplementary Information. [file 41598_2021_94025_MOESM1_ESM.docx]

**Metabolic factors and the risk of Dupuytren’s Disease – data from 30,000 individuals followed for over 20 years**

**Authors**

Mattias Rydberg (1,2), Malin Zimmerman (1,2), Jin Persson Löfgren (1,2), Anders Gottsäter (3), Peter M. Nilsson (4,5) Olle Melander (4,5), Lars B. Dahlin (1,2)

1. Department of Hand Surgery, Lund University, Skåne University Hospital, Malmö, Sweden.
2. Department of Translational Medicine – Hand Surgery, Lund University, Lund, Sweden
3. Department of Vascular Diseases, Lund University, Skåne University Hospital, Malmö, Sweden
4. Department of Emergency and Internal Medicine, Skåne University Hospital, Malmö, Sweden
5. Department of Clinical Sciences, Lund University, Malmö, Sweden

**ORCID**

Mattias Rydberg: 0000-0002-8249-8660

**Corresponding author**

Mattias Rydberg; mattias.rydberg@med.lu.se
Department of Hand Surgery, Skåne university Hospital Malmö, Jan Waldenströms gata 5, S-205 02 Malmö, Sweden; Phone +46 40 33 67 69 (secretary); Fax +46 40 92 88 55

**Supplementary Table S1**

|  | **Model l *** |  |
| --- | --- | --- |
| **Men** | **HR (95% CI)** | **P - value** |
| Diabetes mellitus | 2.03 (1.38 – 3.00) | **< 0.001** |
| Low alcohol consumption | *Reference* | - |
| Moderate alcohol consumption | 1.61 (1.25 – 2.09) | **< 0.001** |
| Heavy alcohol consumption | 2.19 (1.65 – 2.90) | **< 0.001** |
| Normal weight | *Reference* | - |
| Overweight | 0.1 (0.67 – 1.06) | = 0.14 |
| Obesity | 0.65 (0.43 – 0.97) | **= 0.04** |
| **Women** |  | |
| Diabetes mellitus | 2.48 (1.33 – 4.62) | **= 0.004** |
| Low alcohol consumption | *Reference* | - |
| Moderate alcohol consumption | 1.65 (1.13 – 2.41) | **= 0.01** |
| Heavy alcohol consumption | 3.51 (1.92 – 6.41) | **< 0.001** |
| Normal weight | *Reference* | - |
| Overweight | 1.04 (0.76 – 1.43) | = 0.80 |
| Obesity | 0.58 (0.33 – 1.01) | = 0.06 |

**Supplementary Table S1.** Sensitivity analysis, excluding 1303 participants with BMI < 20.0. Sex-stratified Cox regression models with HR for incident DD in relation covariates. * Cox regression model including age, DM, hypertension, smoking, alcohol consumption group, weight group, and manual work. CI; confidence interval, DD; Dupuytren´s Disease, DM; Diabetes Mellitus, HR; Hazard Ratio.

**Supplementary Table S2**

|  | **Model l *** |  |
| --- | --- | --- |
| **Men** | **HR (95% CI)** | **P - value** |
| Diabetes mellitus | 2.23 (1.51 – 3.32) | **< 0.001** |
| Low alcohol consumption | *Reference* | - |
| Moderate alcohol consumption | 1.71(1.31 – 2.21) | **< 0.001** |
| Heavy alcohol consumption | 2.51(1.89 – 3.34) | **< 0.001** |
| Normal weight | *Reference* | - |
| Overweight | 0.82 (0.65– 1.04) | = 0.10 |
| Obesity | 0.61 (0.41 – 0.91) | **= 0.02** |
| **Women** |  | |
| Diabetes mellitus | 2.47 (1.36 – 4.50) | **= 0.003** |
| Low alcohol consumption | *Reference* | - |
| Moderate alcohol consumption | 1.62 (1.11 – 2.36) | **= 0.01** |
| Heavy alcohol consumption | 3.22 (1.76 – 5.89) | **< 0.001** |
| Normal weight | *Reference* | - |
| Overweight | 1.01 (0.73 – 1.39) | = 0.96 |
| Obesity | 0.54 (0.31 – 0.94) | **= 0.03** |

**Supplementary Table S2.** Sensitivity analysis, excluding 7956 participants with a follow-up time ≥ 25 years. Sex-stratified Cox regression models with HR for incident DD in relation covariates. * Cox regression model including age, DM, hypertension, smoking, alcohol consumption group, weight group, and manual work. CI; confidence interval, DD; Dupuytren´s Disease, DM; Diabetes Mellitus, HR; Hazard Ratio.


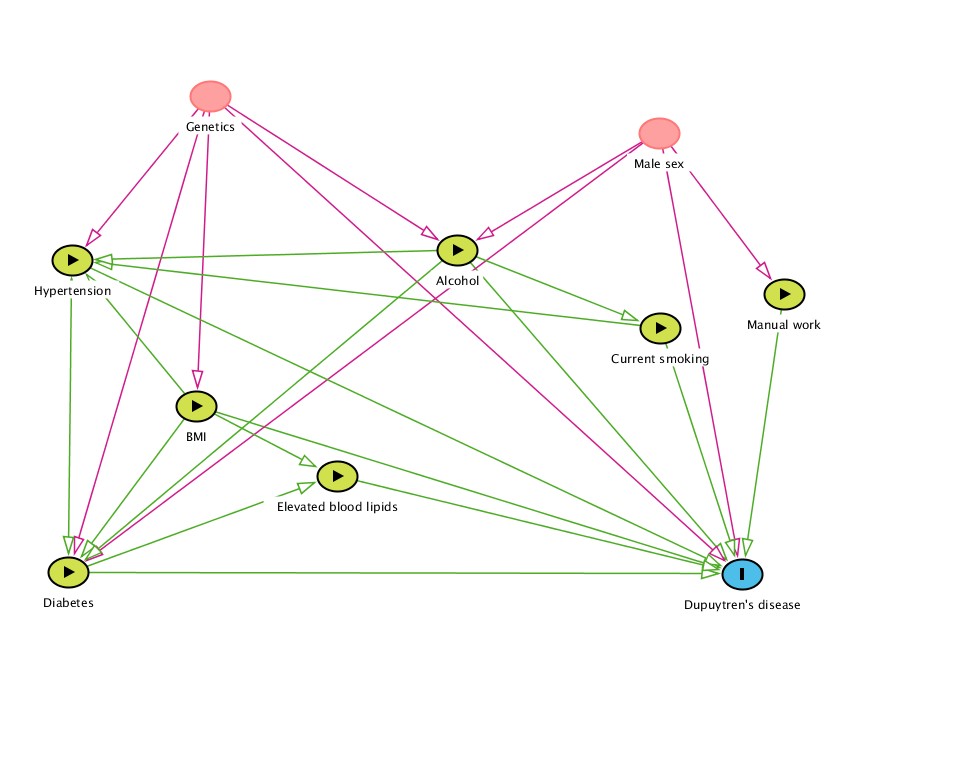


**Supplementary Figure S1.**

Directed acyclic graph (DAG) with arrows representing possible pathological pathways and interactions between exposures, risk factors and DD. (http://www.dagitty.net)
